# Supplementary material for: On the relation between COVID-19, mobility, and the stock market
Source: PLoS One. 2021 Dec 28;16(12):e0261381. doi: 10.1371/journal.pone.0261381 (PMC8714095; doi:10.1371/journal.pone.0261381)
Supplement: S1 Text — (PDF) [file pone.0261381.s001.pdf]

A Tracked Airports

List of tracked airports by ICAO code: ZBAA, OMDB, RJTT, ZSPD, ZGGG, EGLL, LFPG, EHAM, EDDF, LEMD, KATL, KLAX, KORD, CYYZ, MMMX, SBGR, SKBO, SPJC, SCEL, SBSP, YSSY, YMML, YBBN, NZAA
