# Supplementary material for: On the relation between COVID-19, mobility, and the stock market
Source: PLoS One. 2021 Dec 28;16(12):e0261381. doi: 10.1371/journal.pone.0261381 (PMC8714095; doi:10.1371/journal.pone.0261381)
Supplement: Attachment — Submitted filename: Response to Reviewers.pdf [file pone.0261381.s003.pdf]

In this rebuttal letter, the authors respond to each point raised by the academic editor and reviewers.

As indicated by both reviewers, the data was not (yet) made publicly available. In response, we made all data publicly available through figshare. We created a project which includes all data sources used for all figures and statistical analysis. It contains a ".readMe" file containing further explanation. The repository can be accessed through: https://figshare.com/projects/On_the_Relation_between_Covid-19_Mobility_and_the_Stock_Market/126397. The DOI's of each individual dataset are added as references in our revised manuscript.

Reviewer 1

1- I strongly recommend adding a limitation and future research section and explain what was your limitations in research and suggest some offers for future research.

-> We agree. We extended the discussion with further research. Firstly, we suggest to extend the dataset horizon by conducting a comparable study a year from now. This will enable the estimation of seasonal patterns, which is currently not yet possible due to the short horizon of Covid-19. In addition, we suggest further research on the causal relationship between Covid-19 deaths and flights, by analysing these variables on a country level. Lastly, we suggest to explore the weak relationship with Covid-19 cases, on contrast to Covid-19 deaths, by analysing the Covid-19 registration and testing strategies per country.

2-The authors must explain more details about the paper findings and clarify their conclusions for readers.

-> We restructured the discussion and conclusion. Furthermore, we better separated the conclusion from the discussion and added a more clarifying conclusion. The conclusion now better states the strong correlations between covid-19 deaths and mobility and highlights the contrast with the covid-19 cases variable where the correlation is low. Furthermore, we highlighted the lagged variables where covid-19 deaths almost directly influences the mobility variables. Lastly, we added a paragraph based on S11, which presents how mobility recovers after increased covid-19 deaths. It states that traffic rapidly recovers within 5 weeks.

3-The literature needs to be strengthened with prior studies

-> We expanded the literature review of the introduction by adding 8 additional references. Finding publications which relate the stock market to mobility remains challenging.

Reviewer 2

1. Overall, while the direct descriptive method and the quality of the presentation is convincing, from a descriptive point of view, authors do not show deep inferential statistical analysis based on their collected data.

-> We agree, we present an elaborate descriptive analysis followed by a relatively short statistical analysis. However, the authors are of the opinion the work is a contribution on its own. For future work, we could elaborate our study with a deeper statistical analysis.

2 In the introduction section, the authors stated, "The goal is to expose relations between the variables and understand them by using our data". Which variables? Moreover, the authors may clarify the relations more in terms of statistical measures not just graphically.

-> The variables include mobility related ones (vessels, vehicles, flights, train, bicycle), Covid-19 related ones (cases, deaths), and the stock market. We updated the phrasing in the abstract and introduction. In the results section, we do present the relations in terms of Pearson correlation. As mentioned above, we realize this statistical analysis could be elaborated, however, we are of the opinion that the graphical analysis combined with the current statistical analysis is a contribution on its own.

3 In the discussion section, authors should justify why seasonality in the time series is ignored.

-> We agree, we included a paragraph which states that the used data limits us from exploring seasonal relationships. The Covid-19 data is limited to slightly less than a year and both the flight and car data is only available for the past year. Therefore, we could not reliably estimate the impact of seasonal relationships. Estimating these seasonal components would require at least 2 years of historical data.

4 In the discussion section, the authors stated, "Our results show significant relations between most variables. Especially …". Here some statistical evidence is needed. Significance in terms of what?

-> In this paragraph, we refer to the Pearson correlation coefficients between the variables presented in Table 1. We updated the phrasing in this paragraph.
