# Supplementary material for: On the relation between COVID-19, mobility, and the stock market
Source: PLoS One. 2021 Dec 28;16(12):e0261381. doi: 10.1371/journal.pone.0261381 (PMC8714095; doi:10.1371/journal.pone.0261381)
Supplement: Attachment — Submitted filename: paper review_PLOS ONE Journal.docx [file pone.0261381.s002.docx]

1. Overall, while the direct descriptive method and the quality of the presentation is convincing, from a descriptive point of view, authors do not show deep inferential statistical analysis based on their collected data.
2 In the introduction section, the authors stated, “The **goal is to expose relations between the variables and understand them by using our data”**. Which variables? Moreover, the authors may clarify the relations more in terms of statistical measures not just graphically.

3	In the discussion section, authors should justify why seasonality in the time series is ignored.

4	In the discussion section, the authors stated, “**Our results show significant relations between	most variables. Especially … 320**

Here some statistical evidence is needed. Significance in terms of what?
